# English and Chinese Children’s Performance on Numerical Tasks

**DOI:** 10.3389/fpsyg.2018.02731

**Published:** 2019-02-05

**Authors:** Ann Dowker, Anthony M. Li

**Affiliations:** ^1^Department of Experimental Psychology, Oxford University, Oxford, United Kingdom; ^2^Somerville College, Oxford University, Oxford, United Kingdom

**Keywords:** primary school children, mathematical development, number line tasks, number comparison, cross-cultural research, counting system

## Abstract

East Asian pupils have consistently outperformed Western pupils in international comparisons of mathematical performance at both primary and secondary school level. It has sometimes been suggested that a contributory factor is the transparent counting systems of East Asian languages, which may facilitate number representation. The present study compared 35 7-year-old second-year primary school children in Oxford, England and 40 children of similar age in Hong Kong, China on a standardized arithmetic test; on a two-digit number comparison test, including easy, misleading and reversible comparisons; and on a number line task, involving placing numbers in the appropriate position on four number lines: 1–10, 1–20, 1–100, and 1–1000. The Chinese children performed significantly better than the English children on the standardized arithmetic test. They were faster but not significantly more accurate on the Number Comparison and Number Line tasks. There were no interactions between language group and comparison type on the number comparison task, though the performance of both groups was faster on easy pairs than those where there was conflict between the relative magnitudes of the tens and the units. Similarly, there were no interactions between group and number line range, though the performance of both groups was influenced by the range of the number line. The study supports the view that counting systems affect aspects of numerical abilities, but cannot be the full explanation for international differences in mathematics performance.

## Introduction

Recent large-scale cross-national comparisons of mathematical abilities (Askew et al., 2010; Sturman, 2015; Mullis et al., 2016a,b) have shown that East Asian countries like China, Japan, South Korea, and Singapore are usually at the top of international comparisons of mathematics performance. Most studies have found an East Asian advantage in mathematical performance in multiple age groups, starting from preschool (Miller et al., 1995; Geary et al., 1996).

There are many possible reasons for East Asian children’s particularly high performance on these tasks. These include differences in teaching methods: indeed, in recent years, United Kingdom schools have been seeking to develop and use materials and approaches similar to those used in Shanghai and Singapore. Different researchers and policymakers emphasize different aspects of the teaching approaches that they see as beneficial. Some emphasize greater subject knowledge and understanding by East Asian teachers, reinforced by extensive continuous professional development (Ma, 1999); some emphasize greater attempts to foster conceptual understanding (Perry et al., 1993; Stigler et al., 1996; Ma, 1999) some emphasize greater focus on rote learning (Gibb, 2014); some emphasize the ‘mastery’ approach whereby fewer areas within mathematics are covered, but in greater depth, and teachers endeavor to ensure that all pupils in a class have understood one topic before moving on to the next (Jerrim and Vignoles, 2015). Additionally, East Asian pupils often devote more hours per day to mathematics (and some other academic subjects) in school and in homework than those in many other countries. Also, East Asians may value mathematics more than those in many other countries; and appear to place more value on academic achievement in general, and to attribute success more to effort, than many Westerners (Hess et al., 1987; Stigler et al., 1996; Wong et al., 2001).

One further explanation that has been proposed for East Asian children’s relatively high performance in mathematics is that their languages have highly transparent counting systems (Miura et al., 1988). In Chinese, for instance, counting from ten onwards takes the form of A-ten-B, and then A-hundred-B-ten-C. Twelve in Chinese is 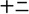 (shi-er), which translates as ten-two; Sixty-two in Chinese is 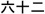 (liu-shi-er), which translates as six-ten-two.

By contrast, the English counting system is more opaque. There are three major irregularities in the English counting system below 100. Firstly, the numbers ‘eleven’ and ‘twelve’ give children no suggestion of the cardinality of the number. In contrast with the Chinese counting words, there are no indications of number values within these English words – that eleven means ten plus one, while twelve means ten plus two. Secondly, the teen words are inverted in relation to with Arabic numerals; e.g., ‘sixteen’ is inverted compared to the Arabic ‘16’ and the Chinese +7 (shi-liu, literally ten-six); the same applies to numbers from thirteen to nineteen. Thirdly, the teen words sound similar to the numbers that are multiples of ten, e.g., ‘sixteen’ sounds like ‘sixty,’ which may create confusion. Even where confusions do not occur, the English counting system does not give as strong clues to the base ten system, as do the counting systems of Chinese and other East Asian languages. This may be important to numerical development for several reasons. It may be easier to learn and remember the counting sequence if it is based on consistent and regular patterns (Miller et al., 1995). It may be easier to understand place value in written arithmetic if it corresponds closely to the oral counting system (Miura et al., 1988). More generally, an oral counting system that is both regular in itself, and transparently related to the written number system may contribute to the precision and accessibility of cognitive representations of number. This idea is a feature of several models of numerical cognition and how it may be influenced by the counting system (Nickerson, 1988; Zhang and Norman, 1995; Zhang and Wang, 2005; Bender and Beller, 2018). Most of these models focus mainly on adult numerical processing, but cross-cultural studies of children have provided some evidence for them.

Some evidence for superior understanding of base 10 and place value in children with highly transparent counting systems comes from work by Miura et al. (1988, 1993), Miura and Okamoto (2003), and Okamoto (2015). They initially investigated the base 10 knowledge on Japanese (transparent counting system) and American (opaque counting system) first graders using Base 10 blocks. These blocks include unit blocks and tens blocks, with the tens blocks having ten segments of units shown on them. The studies revealed that Japanese children were significantly more likely to represent two-digit numbers using a combination of tens blocks and unit blocks, while the American children were more likely just to use unit blocks. This was interpreted as evidence that a transparent counting system facilitates understanding of the semantics of multi-digit numbers by using base-10 knowledge. Follow-up studies were done on different countries and confirmed this difference between the users of transparent and opaque counting systems and (e.g., Miura et al., 1988; Miura and Okamoto, 2003).

One problem with international comparisons is that children in different countries will differ with regard to a wide variety of educational and cultural influences: not just those involving language (Saxton and Towse, 1998). Studies of different language groups in the same country have suggested that language probably affects some specific numerical abilities, but not arithmetic globally. In Wales, most children study in English as elsewhere in the United Kingdom, but about 20% attend Welsh medium schools, where they use the transparent Welsh counting system for arithmetic. However, all schools in Wales follow the same national curriculum. Dowker et al. (2008) investigated the numerical abilities of 6-and year-old children attending English and Welsh medium schools in Wales. They found that there was no difference between the children at the English and Welsh medium schools regarding overall arithmetical performance, but that those in the Welsh medium schools were significantly better at reading and comparing two-digit numbers. Mark and Dowker (2015) studied children in Chinese and English medium schools in Hong Kong. They found that those in the Chinese medium school did perform somewhat better at a standardized arithmetic test, and at backward and forward counting, but, in contrast to the Welsh study, only younger children (6 to 7) and not older children (8 to 9) showed group differences in reading and comparing two-digit numbers.

The superior performance of speakers of languages with regular counting systems on some numerical tasks has led to the question of whether their internal spatial representations of numbers may be more precise. Most commonly, this is studied by means of number line estimation tasks Number line estimation tasks ask participants to indicate an approximate position of a target number within a fixed range on a number line. Siegler and Booth (2004) found that performance on such tasks is related to performance on other numerical tasks, and that it improves with age. Not surprisingly, children find number lines that include a higher number range more difficult than those that involve a relatively low number range: Siegler and Booth (2004) found that they perform better on a 0–10 number line than a 0–20 number line, which is in turn easier for them than a 0–100 number line, while a 1 to 1000 number line is more difficult than any of the previous ones.

Some studies suggest that children using transparent counting systems are better at number line tasks than those using more opaque counting systems, but results are conflicting. Siegler and Mu (2008) found that Chinese kindergarten children performed better than their American counterparts on mental number line estimation tasks involving a number line spanning from 1 to 100. Laski and Yu (2014) found that both Chinese and Chinese-American children performed better than monolingual English-speaking American children, but that children in China performed better on these tasks than Chinese-American children, suggesting that both linguistic *and* educational factors were important. By contrast, Muldoon et al. (2011) did not find such a difference between Chinese and Scottish 4-and 5-year-olds; and indeed when smaller number lines from 0 to 10 and 0 to 20 were included, the Scottish children performed better. This was despite the fact that the Chinese children did do better than the Scottish children on a standardized arithmetic test. Dowker and Roberts (2015) studied children in English and Welsh in Wales, and found a trend for children in Welsh medium schools to be more accurate and quicker on number line tasks, but the difference did not reach significance. However, the Welsh medium children *did* show significantly lower standard deviations than the English medium pupils, indicating more consistency and lower variability in performance.

There are also studies of children, who use counting systems that are *more* opaque than English, such as German, where the oral counting words are systematically inverted with respect to the written counting system, e.g., ‘drei und zwanzig’ (three and twenty) for 23. This might increase the potential for confusing tens and units when translating between the oral and written numbers systems. Such studies have indicated that children who use such counting systems are less accurate in placing numbers on empty number lines children who use counting systems with little or no inversion (e.g., Helmreich et al., 2011; Krinzinger et al., 2011; Klein et al., 2013; Moeller et al., 2015; Bahnmueller et al., 2018a, in press).

The present study focuses on differences between English and Chinese-speaking children. There have already been have been a number of studies comparing numerical performance between these two language groups, as discussed earlier in the introduction. However, such studies have typically investigated either arithmetical performance *or* tasks involving numeral magnitudes *or* number line tasks. It is important to combine arithmetic tests with numeral magnitude and number line tasks, in order to investigate whether Chinese and English speaking children differ in a similar way for all of these tasks, or whether there are some tasks that favor Chinese-speaking children and some that do not. The key aim of the present study was to investigate and compare Chinese and English children’s numerical abilities on all these tasks.

A secondary aim was to look at specific aspects of the tasks that might influence whether, and to what extent, differences are found between Chinese and English children. For example, it is possible that there might be different results for tasks emphasizing different types of symbolic number representation. There are two main types of symbolic number representation that children use: number words and numerals. The numeral notations are transparent and regular in both languages. The number words are much more regular and predictable in Chinese than in English, and as a consequence are also more transparently related to the numerals. One might therefore expect that English children would be mainly disadvantaged in tasks relating to number words: e.g., fast recognition of spoken number words, transcription of number words into numerical notation, and to some degree mental arithmetic. The disadvantage would be expected to be less pronounced in tasks based on numeral notations, such as written arithmetic and symbolic number comparisons. However, this would only be the case if there is a dissociation between representations of numerals and number words. As number word irregularities also decrease the relationship between number words and numerals, they could still affect numeral-based tasks if numeral-based tasks depend in part on translation from number words, or if the two interact.

In this study, we aimed to investigate Chinese- and English- speaking children’s arithmetical abilities. We gave the children a standardized arithmetic test, to check for global differences in arithmetical ability. We also gave them two tasks to measure more specific numerical abilities, which have sometimes been proposed to differ between users of transparent and opaque counting systems. One of these was a two-digit number comparison task, measuring the understanding of place value (Donlan and Gourlay, 1999; Dowker et al., 2008). The other was a task involving placement of visually- presented numbers on empty number lines of different range (Siegler and Booth, 2004; Moeller et al., 2009; Helmreich et al., 2011; Link et al., 2014; Schneider et al., 2018, in press). Both symbolic number comparison (Göbel et al., 2014) and number line task performance (Petitto, 1990; Schneider et al., 2009; Schneider et al., 2018, in press) been found to predict current and future arithmetical performance. Sasanguie et al. (2013) found that both symbolic number comparison and number line task performance in 6-to-8-year-olds predicted their future arithmetical performance, though symbolic number comparison was the strongest predictor. Schneider et al. (2018, in press) carried out a meta-analysis, which also indicated that both symbolic number comparison and number line task performance predicted arithmetical performance, but suggested that number line task performance was the strongest predictor in 6-to-9-year-olds, and that the two types of task were equally strong predictors in older children.

We predicted that the Chinese pupils would perform better in the standardized arithmetic test, on the basis that in general, Chinese pupils perform better than English pupils in most comparisons of mathematical performance, and in particular, Mark and Dowker (2015) found that Chinese pupils performed better than English pupils on the same standardized arithmetic test.

We predicted more tentatively that they would do better on the number comparison task, as this had been found for Welsh- versus English-speaking children (Dowker et al., 2008), and Chinese versus German children (Lonneman et al., 2016), though not in Mark and Dowker (2015) study of Chinese-speaking versus English-speaking children. There is also evidence that performance on two-digit number comparison tasks is affected by other aspects of counting systems, such as the inversion property of some languages including German (Nuerk et al., 2005) and the vigesimal structure of numbers over 60 in French (Van Rinsveld and Schiltz, 2016).

In addition, we predicted that English children and Chinese children might be differentially affected by the difficulty of the comparison type. Following Donlan and Gourlay (1999), the number comparison task included three different types of comparison. Transparent comparisons were those that did not involve any conflict between the relative values of the decades and the units. Either the numbers shared a unit value lower than either decade value (e.g., 21 vs. 71), or both comparisons contained repeated digits (e.g., 33 vs. 88). Misleading comparisons were those where the unit values differed in the opposite direction from the decade values: e.g., 32 vs. 29. Reversible comparisons were similar to misleading comparisons, but specifically involved pairs where each number reversed the decade and unit values of the other: e.g., 91 vs. 19. We predicted that the Chinese and English children would show greater differences in speed and accuracy for comparisons involving misleading or reversible comparisons than for transparent comparisons. This was because, if Chinese children have more solid representations of two-digit numbers, they would be better able to focus just on comparing the tens and to resist interference from the relative values of the units; and that this would show up mainly in comparisons where there is a conflict between the relative values of the decade numbers and the unit numbers.

We also predicted that the Chinese-speaking children would be more accurate and faster on the number line tasks, due to a greater understanding of, and facility with, multi-digit numbers and their relative magnitudes. While there have been a number of studies of Chinese children’s number line task performance, for the most part such studies have not, to our knowledge, examined differences between different number line ranges, with the notable exception of Siegler and Mu (2008) study, and that only looked at preschoolers with limited experience of the larger number line ranges. This made it important to investigate whether number line range had similar or different effects on Chinese and English children. We predicted that *both* groups of children would find the number lines with larger number ranges would be more difficult for than those with smaller number ranges, and would thus show lower accuracy scores and higher reaction times for the number lines with the larger ranges. However, we also predicted that the differences between Chinese and English-speaking children would be greater for number lines with ranges of 0–20 or more than for the 0–10 number line, because the greater transparency of the Chinese counting system only comes into play for numbers over 10. Thus, any advantages to children of using the more transparent Chinese counting system would be expected to emerge only at the point where their counting system *does* become more transparent than the English counting system.

Thus, we expected that combining the standardized arithmetic test, the number comparison task and the number line task would shed light on what aspects of numerical processing are most influenced in this age group by cultural differences, and on whether any such differences are readily explainable in terms of differences in internal representation of numbers, or are better explained in other ways.

## Materials and Methods

### Participants

Seventy five children (30 girls) participated in the study. They were tested at the end of the first term of their second year of primary school. They included 35 children (10 girls, 25 boys) attending primary schools in Oxford, and 40 (20 girls, 20 boys) attending primary schools in Hong Kong. The mean age of the children was 7.2 years (*SD*: 0.77). The English children had a mean age of 7.09 years (*SD*: 0.95) and the Chinese children 7.3 years (*SD*: 0.56). There was no significant age difference between the two language groups, as confirmed by an independent-samples *t*-test [*t*(73) = 1.204; *p* = 0.56; Cohen’s *d* = 0.26]. All of the Oxford children spoke English as their first language, and none had any knowledge of Chinese or any other East Asian language. All of the Hong Kong children spoke Cantonese as their first language. Most had had some limited exposure to English, but all were taught their main school subjects, including mathematics, in Chinese.

Ethical approval for the study was obtained from Oxford University’s Central University Research Ethics Committee; and informed written parental consent was obtained for all participants.

### Procedure

All participants were given the same tests in the same order: the standardized arithmetic test, followed by the number comparison test, followed by the number line estimation test. The standardized arithmetic test was completed with pencil and paper, and the other tests were given on a Lenovo G50 laptop. Instructions were given to the children in their native language by a bilingual experimenter. Participants were tested individually in a quiet room in their schools. The whole testing session lasted for approximately 40 min.

### Standardized Arithmetic Test

Participants were given the British Abilities Scales (BAS) 2^nd^ edition Basic Number Skills test (Elliott et al., 1996), designed to assess the numerical abilities of children between the ages of 6 and 16. The assessment consists of a series of questions, split into different sections which increase in difficulty as the children progress. Most of the questions involve written calculation. All of the four arithmetical operations are included. There are 46 items in total, arranged in six blocks (A to F); the first four blocks consist of eight items each, and the last two blocks have seven items each. The test is stopped when the child makes four or more errors within a section. In practice, no child progressed further than Section D.

The first section, Section A, includes four numbers that children are asked to read aloud: 100, 12, 40, and 31. It also includes four written arithmetic problems, presented in vertical form: 2 + 3, 4 – 1, 9 + 5, and 18 − 5. The second section, Section B, includes two numbers that children are asked to read aloud: 215 and 370. It also includes a request to point out the orally presented number ‘five hundred and ninety four’ out of five written numbers ranging from 54 to 50094. It includes five written arithmetic problems, presented in vertical form: 15 + 23; 2 × 4; 17 − 5; 13 + 99; and 38 + 57.

The third section, Section C, includes eight written arithmetic problems: two involving multiplication of a two-digit number by a single-digit number; three involving division of two-digit numbers by single-digit numbers; two two-digit subtractions requiring borrowing; and one involving addition of decimals (45.01 + 57.89).

The fourth section, Section D, includes eight written arithmetic problems. These include one problem involving addition of fractions (1/8 + 1/4); one subtraction of fractions (2/3 − 1/3); two problems involving writing decimals as percentages; one problem involving division of a two-digit number by a smaller two-digit number; one problem involving division of a three-digit number by a two-digit number; one involving multiplication of two two-digit numbers; and one involving decimal arithmetic (3(2.7 + 9.3)).

The fifth and sixth sections, Sections E and F, will not be described as no child reached these sect.

### Number Comparison Test

Children were given Donlan and Gourlay (1999) number comparison test. The task was slightly modified in order for it to be used with current computer systems.

There were three types of number pair stimuli – transparent, misleading and reversible. Transparent stimuli were defined as number pairs that varied in the number of tens but had the identical number of units (e.g., 91 and 71) or with repeated digits (e.g., 55 and 44). Participants could make judgments for the response by only looking at the tens. Misleading stimuli are number pairs with a higher number of digits in the smaller two-digit number than that in the bigger one (e.g., 31 and 27). Judgments to these stimuli require holistic processing of both tens and units for correct comparison. Reversible pairs included number pairs with opposite tens and digit positions (e.g., 64 and 46). The items were presented in a new random order for each participant, and were not presented in blocks. Error scores and reaction times were the main measures of the task. The full set of stimuli is displayed in Table 1.

**Table 1:** Number comparison task (adapted from Dowker et al., 2008).

Transparent	Misleading	Reversible
73 43	51 47	85 58
66 55	61 18	61 16
54 94	53 39	14 41
70 10	62 59	56 65
96 86	27 42	43 34
11 99	71 91	76 67
60 50	17 51	39 93
71 91	43 38	25 52
44 55	27 31	46 64

EPrime 2.0 was used to present pairs of numbers side by side on the laptop. The viewing distance was 60 cm. The presentation sequence consisted of a fixation point (500 ms), followed by a slide with two two-digit numbers presented side by side, of approximately 5 cm apart. The number slides only changed to a fixation screen after the laptop detected a response. The whole process was repeated for the remaining trials.

When pairs of two-digit numbers were presented on each slide, participants were asked to give responses on the keyboard by indicating whether the number on the left or the number on the right was the larger number. Before starting, participants were instructed to give responses by two keys on either ends of the keyboard, and their response (left or right key) was consistent to their opinions of where the larger numbers were (left or right key respectively). To prevent contradiction with comparing the physical sizes of stimuli, subjects were given three practice trials to familiarize themselves with the equally-sized numbers.

### Number Line Estimation Test

The children were given four number line estimation tasks (0–10, 0–20, 0–100, and 0–1000) in that order. The test was based on that used by Siegler and Booth (2004), and the sets were those used by Moeller et al. (2011). Once again, in this study, the tasks were carried out on a laptop screen, with the program devised using EPrime 2.0. The number line was presented, at the bottom of the screen, as a long green horizontal rectangle of length 16.8 cm and width 2.4 cm. The ends of the number line were clearly shown (font size: 70) on both sides of the rectangle – 0 on the left; 10/20/100/1000 on the right, depending on the task. Target numbers were presented visually at the center of the screen (font size: 80) one at a time. Before the start of the test, each child was given a couple of practice trials in which they were asked to point to the approximate positions of 5 and 8 on a 0–10 number line. The aim was to check if the children understood the meanings of 0 and 10 at either ends of the rectangle. If the participant demonstrated that they understood that 8 was on the right of 5, the experimenter said, ‘Well done. Now let’s get on to the real thing.’ All children used the pointer of a mouse to give response by clicking on the various positions on the rectangle. The rectangle was designed to appear continuous, but was segregated into 100 slices after a response was given. The respective rectangle that was hit was recorded as a percentage response on the number line. The main measure of the tasks was the percentage difference between the response value on the rectangle and target number (percentage absolute error; henceforth abbreviated to PAE). After each response, there was a 1000ms-delay. Responses made outside the area of the rectangle were not detected by the program, and therefore the child would be reminded to respond again inside the rectangle. There were 10 trials each for 0–10 and 0–20 tasks, and 19 trials each for 0–100 and 0–1000 tasks. On each number line, the order of the target numbers to be estimated was randomized across all children.

The 10 target numbers on the 0–10 number line were 6, 0, 7, 2, 8, 1, 4, 9, and 3. The 10 target numbers on the 0–20 number line were 10, 12, 1, 13, 4, 15, 19, 7, 27, and 5. The 19 target numbers on the 0–100 number line were 50, 27, 2, 64, 35, 7, 13, 99, 75, 47, 3, 11, 82, 95, 9, 17, 6, 18, and 53. The 19 target numbers were 500, 4, 96, 465, 287, 989, 26, 432, 173, 823, 87, 124, 367, 679, 57, 107, 73, 92, and 725.

There was no set time limit, but children were asked to respond as quickly as possible, overt use of strategies other than estimation (such as counting) was discouraged. The scoring measures used were Percentage of Absolute Error (PAE), and also reaction time, as used e.g., by Schneider et al. (2009).

## Results

The data were entered and analyzed using SPSS Statistics 22 (SPSS, Inc. 2013).

### Group Comparisons

#### Standardized Arithmetic Test

The mean raw score on the arithmetic test was 16.4 (*SD* = 4.6). The Chinese children obtained a mean score of 18.35 (*SD*: 3.51). The English children obtained a mean score of 14.17 (*SD* = 4.72). An independent samples *t*-test showed that this difference was significant [*t*(73) = 4.39; *p* < 0.01; Cohen’s *d* = 1.02], with the Chinese children performing significantly better than the English children.

#### Number Comparison Test

A repeated-measures ANOVA was carried out with Comparison type (Easy vs. Misleading vs. Reversible) as the within-participants variable Language Group (English versus Chinese) as the grouping factors; and Number Comparison score as the dependent variable. Though there was a trend toward greater accuracy by Chinese than English children, the group difference did not reach significance [*F*(1,73) = 2.86; *p* = 0.068; $\eta_{p}^{2}$ = 0.209]. There was no significant within-participants effect [*F*(2,146) = 1.075; *p* = 0.303; $\eta_{p}^{2}$ = 0.01], nor any significant interaction effect between Language Group and Number Comparison score [*F*(2,146 = 0.8; *p* = 0.449; $\eta_{p}^{2}$ = 0.011].

Another repeated-measures ANOVA was carried out with Comparison type (Easy vs. Misleading vs. Reversible) as the within-participants variable; Language Group (English versus Chinese) as the grouping factors; and Reaction Time score in milliseconds as the dependent variable. There was a strong between-participants effect of Language Group [*F*(1,73) = 50.374; *p* < 0.001; $\eta_{p}^{2}$ = 0.415]. Chinese children were much faster than English children. There was also a significant within-participants effect [*F*(2,146) = 7.352; *p* = 0.001; $\eta_{p}^{2}$ = 0.094]. Pairwise comparisons showed that reaction times were longer for Misleading than Reversible problems, and for both Misleading and Reversible problems than Easy problems. There was, however, no significant interaction effect between Language Group and Comparison type [*F*(2,146) = 0.95; *p* = 0.389; $\eta_{p}^{2}$ = 0.013]. Thus, the language groups differed in overall performance, but not with regard to the relative difficulty of the comparison types.

#### Number Line Tasks

The Number Line Mean Reaction Times in milliseconds are also given in Table 2. There was a significant within-participants effect of Number Line Range [*F*(3,219) = 15.114, *p* < 0.001; $\eta_{p}^{2}$ = 0.186]. Pairwise comparisons showed no significant difference in Mean Reaction Time between the 0–20 and the 0–1000 number lines (*p* = 0.47) and only a trend toward significance between the 0–100 and 0–1000 number lines (*p* = 0.084), but all other differences between number lines were significant. The difference between the 0–20 and the 0–100 number lines reached significance (*p* = 0.031) and the differences between the 0–10 and the 0–20 number lines; the 0–10 and the 0–100 number lines; and the 0–10 and the 0–1000 number lines were all highly significant (*p* < 0.001). There was a significant between-participants effect of Language Group [*F*(1,73) = 12.69; *p* < 0.001; $\eta_{p}^{2}$ = 0.161). However, there was no significant interaction between Language Group and Number Line Range [*F*(3,219 = 1.28; *p* = 0.283; $\eta_{p}^{2}$ = 0.161].

**Table 2:** Mean scores (out of 9) and RTs (in milliseconds) by each language group on easy (transparent), misleading, and reversible number comparison items.

Number	Language group		
comparison			
item type	Chinese (*N* = 40)	English (*N* = 35)	Combined (*N* = 75)
**Scores**			
Easy	8.75 (0.49)	8.71 (0.67)	8.73 (0.58)
Misleading	8.68 (0.57)	8.69 (0.58)	8.68 (0.57)
Reversible	8.95 (0.22)	8.77 (0.49)	8.87 (0.39)
Total score^∗^	26.38 (0.90)	26.12 (1.00)	26.26 (1.04)
**RTs**			
Easy	1512.25 (68.43)	3443.9 (1816.32)	2285.46 (1613.64)
Misleading	1657.78 (678.4)	3775.83 (1838.64)	2606.21 (1690.46)
Reversible	1697.71 (650.28)	3885.9 (1704.23)	2664.29 (1633.52)
All problems	1622.58 (612.47)	3678.54 (1705.68)	2551.99 (1599.31)

*RT, reaction time. Standard deviations are given in brackets. ^∗^Total score is out of 27.*

For each participant the mean PAE score for each number line was calculated. The PAE score of each trial was the absolute distance between the true position of the target number and the response. Table 3 gives the mean PAE and reaction time for each number line in each language group. Two repeated-measures ANOVAs were conducted with Number Line Range (0–10 vs. 0–20 vs. 0–100 vs. 0–1000) as the within-participants factor; Language Group (English versus Chinese) as the grouping factors, and Mean PAE and Mean Reaction Time as dependent variables.

**Table 3:** Mean percentages of absolute error (PAE) and RTs (in milliseconds) by each language group on the number line tasks.

Number line	Language group		
	Chinese (*N* = 40)	English (*N* = 35)	Combined (*N* = 75)
**PAE**			
0–10	7.1 (3.67)	8.21 (4.13)	7.61 (3.90)
0–20	7.23 (4.34)	5.68 (2.48)	6.52 (3.67)
0–100	9.25 (5.22)	9.78 (6.61)	9.5 (5.86)
0–1000	17.3 (9.12)	16.6 (11.15)	16.98 (10.04)
**RTs**			
0–10	5060.05 (1893.28)	6803.44 (3091.47)	5829.19 (2622.35)
0–20	4204.6 (1746.47)	5233.31 (1701.35)	4658.44 (1728.58)
0–100	3795.68 (1745.45)	4912.48 (2035.38)	4289.39 (1946.19)
0–1000	3890.19 (1348.24)	5663.88 (2734.88)	4672.7 (2242.44)

*RT, reaction time. Standard deviations are given in brackets.*

For Mean PAE, there was a significant within-participants effect of Number Line Range [*F*(3,219) = 68.06; $\eta_{p}^{2}$ = 0.49]. Pairwise comparisons showed no significant difference in Mean PAE between the 0–10 and 0–100 number lines (*p* = 0.15) but all other comparisons were significant. The mean difference in PAE between the 0–10 and the 0–20 number line reached significance (*p* = 0.031) and the differences between the 0–10 and the 0–1000 number lines; the 0–20 and the 0–100 number lines, the 0–20 and the 0–1000 number lines; and the 0–100 and the 0–1000 number lines were all highly significant (*p* < 0.001). There was no significant effect of Language Group [*F*(1,73) = 0.021; *p* = 0.895; $\eta_{p}^{2}$ = 0). Nor was there any significant interaction between Language Group and Number Line Range [*F*(3,219) = 0.899; *p* = 0.443; $\eta_{p}^{2}$ = 0.012].

## Discussion

Overall, the results supported the hypotheses that Chinese children would perform better on tests of numerical abilities, but this varied to some degree with the measures used. The Chinese children performed better on a standardized arithmetic test. They were faster but not more accurate on a number comparison task; though near-ceiling effects might have contributed to the lack of group differences in accuracy. They had significantly faster reaction times to the number line tasks, but did not differ significantly in accuracy, which in this task cannot be attributed to ceiling effects.

The better performance by Chinese than English children in the standardized arithmetic test was in line with our predictions, and similar to findings in many other studies (e.g., Mark and Dowker). This is likely to be due to several factors, which may include the transparency of the counting system; the greater length of time devoted to arithmetic in Chinese schools; stronger societal value placed on mathematics in China; and possibly differences in teaching methods. The superior performance by Chinese children is particularly striking in view of the fact that the test was developed and standardized in Britain, making it very unlikely that Chinese children would have had any direct preparation for it.

The prediction that the Chinese children would do better than the English children in number comparison tasks was partially supported. They were faster, but did not differ in accuracy. Their faster reaction times give some support to Miura et al. (1988) hypothesis that the use of transparent counting systems may improve understanding of the semantics of the base ten system, and are in line with Dowker et al. (2008) findings comparing English and Welsh children, and Lonneman et al. (2016) findings comparing Chinese and German children. This result suggests that certain multi-digit number tasks are indeed easier for children who speak languages with transparent counting systems. The lack of group differences in *accuracy* may not go against this hypothesis, given the near-ceiling effects for accuracy, mentioned above; and also because of the possibility of a speed-accuracy trade-off. However, the results do not confirm the prediction that there would be an interaction between group and comparison type, and thus do not support a view that the Chinese and English children are likely to be using fundamentally different strategies, or to have fundamentally different number representations. Both groups were faster at comparing easy (transparent) pairs than misleading pairs, with reversible pairs coming in between. The fact that the reversible pairs were somewhat easier than the other misleading pairs may be due to the fact that fewer numbers needed to be kept in working memory. However, the difference was not great: the misleading and reversible pairs were more similar to one another than they were to the transparent pairs, supporting earlier findings by Nuerk et al. (2005). Contrary to the predictions, English children were not more affected than the Chinese children by the comparison type.

The results also give partial, but not total, support for the hypothesis that children, who use a transparent counting system, would be better at placing numbers on an internal number line. Once again, the Chinese children were faster, but they were not more accurate. Again, a speed-accuracy trade-off may have contributed to the results. It should be noted that in this case, different cultural influences may have been in conflict. The Chinese children had a more transparent counting system, and may also have been subject to other positive educational influences; but the English children had more specific experience with number lines.

Number lines play a significant part in United Kingdom mathematics instruction. The United Kingdom national curriculum for primary school mathematics^1^ indicates that pupils are expected to be taught to use number lines throughout years 1 to 6, with increasing levels of sophistication. This In part related to an emphasis in the United Kingdom on mathematical estimation in general. On the other hand, a careful scan of the HK’s primary school curriculum reveals no mention of either ‘number estimation’ or ‘number line’^2^. The focus of teaching in HK appears to be more geared toward instruction in procedures for exact mental and written calculation. Although systematic quantitative data are still needed, brief interviews with the children indicated that the United Kingdom pupils had had practice with the use of number lines at school, while most Hong Kong pupils reported a lack of experience with them. The Hong Kong pupils tended to respond to the number line tasks by utilizing strategies for counting exact quantities by trying to visualize imaginary counters on the stimulus, without taking much notice of the extremes of the number line; and verbalized counting far more than the United Kingdom pupils did. This was inferred from consistent patterns of verbalization of counting in the HK sample but not in the United Kingdom sample.

The number line range had significant effects on performance by both groups, supporting findings by Siegler and Booth (2004) and others. Number lines with larger ranges were generally more difficult, in that they elicited larger errors. There was little difference between performance on the 1–10 and the 1–20 number line, but the PAE increased with increasing number line range beyond 20. Reaction time on the other hand actually decreased from the 1 to 10 number line to those with higher ranges, though this effect showed signs of reversal for the number line with a 1–1000 range. This may be in part due to practice effects, as the 1 to 10 number line was given first, and possibly fatigue on the 1–1000 number line. It may also reflect changes in strategy, with a reduction in counting-related strategies as the number line range increased.

The fact that there was no interaction between group and number line range, with regard to either accuracy or reaction time, suggests that the English and Chinese children were not using fundamentally different strategies for the number line tasks. It would be desirable in future studies to investigate and compare the strategies of English and Chinese children directly, perhaps combining the strategy analyses of Link et al. (2014) with the eye tracking measures developed by Schneider et al. (2018).

Thus, the study supports the view that the transparency of a counting system influences some but not all numerical abilities. It is important to remember that the counting system is by no means the only reason for cultural and national differences in mathematics. As already mentioned, such differences are influenced by educational methods and by cultural attitudes to education. When children, who use different counting systems, receive the same curriculum, they tend to perform similarly on arithmetic tests, though often differing in more specific numerical abilities (Dowker et al., 2008; Dowker and Roberts, 2015). Thus, it is most likely that the differences in performance on the arithmetic test in the present study were due to educational and/or other cultural factors, while the differences on other numerical abilities may more likely to have been due to linguistic factors.

There is a caveat to be made here: since the group differences were found for reaction time but not for accuracy, it is possible that they reflect differences in speed of responses to tasks in general, rather than numerical tasks in particular. Chinese children may either have a generally faster speed of processing, or be more likely to interpret test situations as requiring speedy responses. Because of a possible speed-accuracy trade-off, a greater Chinese emphasis on speed could have led to an underestimation of differences in ability to produce accurate responses. Future studies should include non-numerical control tasks, to check for this possibility. Also, even if the effects are based on the counting system, they might reflect not the greater transparency of the Chinese counting system, but the relative shortness and faster pronounceability of Chinese number words (Ellis and Hennelly, 1980). This possibility could be tested in the future by making direct comparisons between Chinese- and Welsh-speaking children, as their counting systems are similarly transparent, but Welsh number words are *longer* than English number words.

Further studies are needed to investigate the relative importance of linguistic, educational and other cultural factors Such studies should if possible include investigations of specific educational content, such as the use of number lines, and cultural factors such as differences in finger counting techniques (Göbel et al., 2011). Also, future studies should incorporate larger samples with a wider variety of ages, languages and backgrounds. One potential limitation of the present study is that there was relatively limited information about possible social and economic differences between the groups. The backgrounds appeared to be similar (varied but predominantly middle-class); but quantitative information on this matter was not collected. This should be investigated more systematically in future research.

Most research on cross-linguistic effects on arithmetic has focused on the effects of counting system transparency. The present study has combined investigation of standardized test performance with investigation of more basic numerical abilities, and indicates that counting system transparency does indeed have some effect on both. Future studies should now look more at other linguistic differences that might affect arithmetic and number processing (Göbel et al., 2011; Dowker and Nuerk, 2016; Bahnmueller et al., 2018b). These include, for example, phonological factors such as the length and pronunciation speed for number words; grammatical factors such as whether a language has dual and plural markers; and semantic factors such as the ways in which numerical concepts such as ‘few,’ ‘many,’ ‘more,’ and ‘less’ are represented in words and symbols.

Future studies should also include measures of domain-general factors, such as IQ, working memory, and verbal and spatial ability, which could directly influence arithmetical and numerical abilities, and possibly also mediate or moderate relationships between numerical abilities and arithmetic. Research is providing increasing evidence for the role played by such domain-general factors in numerical development (Schneider et al., 2008; Schneider et al., 2018, in press). For example, Simms et al. (2016) have found that visuospatial and visuomotor abilities explain much of the relationships between number line task performance and arithmetic in 8-to 10-year-olds; though they also found that PAE (unlike some other number line performance measures) predicted arithmetic even after controlling for visuomotor and visuospatial abilities. Other researchers have found that number line performance is correlated with domain-general spatial abilities (Gunderson et al., 2012); measurement skills (Cohen and Sarnecka, 2014) and overall IQ (Schneider et al., 2009). It is important to investigate whether these and other domain-general abilities show similar relationships to numerical abilities in different language groups.

A potential limitation is that the tasks, including the number line tasks, were given in a fixed order. This was done, so as to avoid the need to use presentation order as an additional variable; but it makes it difficult to draw conclusions as to whether the lower reaction times to lines with higher number ranges were due to practice effects or to strategy changes. Future studies should look at whether there are order effects.

The present study adds to our knowledge base about cultural differences in numerical abilities, by demonstrating that Chinese and English children do indeed appear to show differences in numerical tasks as well as in formal arithmetic. The Chinese children were much more accurate than the English children in the formal arithmetic test. They did not show such differences in accuracy in the non-arithmetical numerical abilities. However, they did show striking differences in *speed*: the Chinese children were much faster than the English children on both the number comparison task and the number line task.

The results do not support the study’s secondary prediction that the differences would affect tasks involving number words but not those involving numerical notation. The number line tasks involved numbers presented *only* in numerical notation, and not through spoken words; and yet group differences were found. This suggests that, at least with children at this age, numerical notations and number words may not be processed totally independently. However, we need to be cautious in making strong interpretations of these results, since the main purpose of the study was not to compare numerical notations with spoken words, and they were not systematically varied.

One possible reason for the findings that group differences were stronger for arithmetic than for accuracy (though not speed) on non-arithmetical tasks is that the arithmetic problems might have relied more on verbal processes, while the number line and number comparison problems might have relied more on visuospatial processes. The transparency of the verbal counting system would be likely to have a greater effect on verbal than visuospatial processes. To solve arithmetic problems, the children might have relied at least partially on verbal processes that might account for the differences between groups. Verbal processes might have been slightly more efficient with more transparent verbal number words (i.e., Chinese). On the contrary, number lines would be rather tap into visa-spatial processes and an internal number representation without any need of verbal processing and, by consequence, produce reduced differences between the groups. In brief, the differences between the groups might emerge when the numerical tasks involve number words at the processing level (even though the task material itself is not presented in a verbal format), such as arithmetic typically.

There are numerous educational and cultural differences between Chinese and English children that are likely to contribute to the results. It is, however, likely that the counting system is a significant contributory factor, as some other studies have found differences between users of transparent and non-transparent counting systems even within the same geographical region and educational system (Dowker et al., 2008; Mark and Dowker, 2015) and even between performance by the same individuals using different counting systems within the Czech language (Pixner et al., 2011). The results, however, do not indicate that Chinese and English children have fundamentally different internal representations of number, though this may depend on age, and findings might be different for older or younger children. It is perhaps more likely that a transparent counting system facilitates arithmetical and numerical performance by making the numerical characteristics of, and the relationships and differences between, two-digit numbers more salient, and by reducing the load that multi-digit numbers place on working memory.

## Ethics Statement

The study was carried out in accordance with the guidelines of the Central University Research Ethics Committee of Oxford University. As the study involved work with children, written parental consent was obtained for all participants, using an opt-in procedure, where parents were given an information sheet and signed a consent form. All aspects of the study were carried out in accordance with the university’s Inter-Divisional Research Ethics Commitee’s Protocol 25, which sets out the expected procedures for work with children in schools. The project was approved by the Central University Research Ethics Committee of Oxford University.

## Author Contributions

AD provided the tests and took the main role in writing this article. AML carried out the experiments and did all necessary translations. AD and AML worked together in designing the project and carrying out the statistical analyses.

## Conflict of Interest Statement

The authors declare that the research was conducted in the absence of any commercial or financial relationships that could be construed as a potential conflict of interest.

## Footnotes

1 National curriculum in England: framework for key stages 1 to 4 (effective from 1 September, 2015 to 31 August, 2016) – https://www.gov.uk/government/publications/national-curriculum-in-england-framework-for-key-stages-1-to-4/the-national-curriculum-in-england-framework-for-key-stages-1-to-4.

2 Contents of Curriculum, Learning Targets of Key Stages 1 and 2 –http://www.edb.gov.hk/attachment/en/curriculum-development/kla/ma/curr/ chapter%204_1.pdf.

## References

[B1] Askew M. Hodgen J. Hossain S. Bretscher N. (2010). *Values and Variables: Mathematics Education in High-Performing Countries.* London: Nuffield Foundation.

[B2] Bahnmueller J. Maier C. Goebel S. Moeller K. (2018a). Direct evidence for linguistic influences in two-digit number processing. *J. Exp. Psychol. Learn. Mem. Cogn.* 10.1037/xlm0000642 [Epub ahead of print]. 30024256

[B3] Bahnmueller J. Nuerk H. C. Moeller K. (2018b). A taxonomy proposal for types of interactions of language and place-value processing in multi-digit numbers. *Front. Psychol.* 9:1024. 10.3389/fpsyg.2018.01024 29988596 PMC6026795

[B4] Bender A. Beller S. (2018). “Numeration systems as cultural tools for numerical cognition,” in *Mathematical Cognition and Learning: Language and Culture in Mathematical Cognition*, eds Berch D. B. Geary D. C. Koepke K. Mann (Cambridge, MA: Academic Press), 297–320. 10.1016/B978-0-12-812574-8.00013-4

[B5] Cohen D. J. Sarnecka B. W. (2014). Children’s number-line estimation shows development of measurement skills (not number representations). *Dev. Psychol.* 50 1640–1652. 10.1037/a0035901 24512172 PMC5087800

[B6] Donlan C. Gourlay S. (1999). The importance of nonverbal skills in the acquisition of place-value knowledge: evidence from normally-developing and language-impaired children. *Br. J. Dev. Psychol.* 17 1–19. 10.1348/026151099165113

[B7] Dowker A. Bala S. Lloyd D. (2008). Linguistic influences on mathematical development: how important is the transparency of the counting system. *Philos. Psychol.* 21 523–538. 10.1080/09515080802285511

[B8] Dowker A. Nuerk H.-C. (2016). Linguistic influences on mathematics. *Front. Psychol.* 7:1035. 10.3389/fpsyg.2016.01035 27462286 PMC4940406

[B9] Dowker A. Roberts M. (2015). Does the transparency of the counting system affect children’s numerical abilities? *Front. Psychol.* 6:945. 10.3389/fpsyg.2015.00945 26217270 PMC4493320

[B10] Elliott G. Murray D. Pearson L. (1996). *British Abilities Scales*, 2nd Edn. Windsor: National Foundation for Educational Research.

[B11] Ellis N. C. Hennelly R. A. (1980). A bilingual word-length effect: Implications for intelligence testing and the relative ease of mental calculation in Welsh and English. *Br. J. Psychol.* 71 43–52. 10.1111/j.2044-8295.1980.tb02728.x

[B12] Geary D. C. Salthouse T. A. Chen G. Fan L. (1996). Are East Asian versus American differences in arithmetical ability a recent phenomenon? *Dev. Psychol.* 32 254–262. 10.1037/0012-1649.32.2.254

[B13] Gibb N. (2014). Rote learning will bring pupils ‘on par with peers in Far East’. Available at: https://www.telegraph.co.uk/education/educationopinion/10645954/Rote-learning-will-bring-pupils-on-par-with-peers-in-Far-East.html

[B14] Göbel S. M. Shaki S. Fischer M. H. (2011). The cultural number line: a review of cultural and linguistic influences on the development of number processing. *J. Cross Cult. Psychol.* 42 543–565. 10.1177/0022022111406251

[B15] Göbel S. M. Watson S. E. Lervag A. Hulme C. (2014). Children’s arithmetic development: it is number knowledge, not the approximate number sense, that counts. *Psychol. Sci.* 25 789–798. 10.1177/0956797613516471 24482406

[B16] Gunderson E. A. Ramirez G. Beilock S. L. Levine S. C. (2012). The relation between spatial skill and early number knowledge: the role of the linear number line. *Dev. Psychol.* 48 1229–1241. 10.1037/a0027433 22390659 PMC10811729

[B17] Helmreich I. Zuber J. Pixner S. Kaufmann L. Nuerk H.-C. Moeller K. (2011). Language effects on children’s non-verbal number line estimations. *J. Cross Cult. Psychol.* 42 598–613. 10.1177/0022022111406026

[B18] Hess R. D. Chang C.-M. McDevitt T. M. (1987). Cultural variations in family beliefs about children’s performance in mathematics: comparisons among people’s republic of China, Chinese-American, and Caucasian-American families. *J. Educ. Psychol.* 79 179–188. 10.1037/0022-0663.79.2.179

[B19] Jerrim J. Vignoles A. (2015). *The Causal Effect of East Asian ‘Mastery’ Teaching Methods on English Children’s Mathematics Skills.* DoQSS Working Paper No. 15-05. London: University College London Institute of Education.

[B20] Klein E. Bahnmueller J. Mann A. Pixner S. Kaufmann L. Nuerk H. C. (2013). Language influences on numerical development—inversion effects on multi-digit number processing. *Front. Psychol.* 4:480. 10.3389/fpsyg.2013.00480 23935585 PMC3733006

[B21] Krinzinger H. Gregoire J. Desoete A. Kaufmann L. Nuerk H. Willmes K. (2011). Differential language effects on numerical skills in second grade. *J. Cross Cult. Psychol.* 42 614–629. 10.1177/0022022111406252 11699686

[B22] Laski E. V. Yu Q. Y. (2014). Number line estimation and mental addition: examining the potential roles of language and education. *J. Exp. Child Psychol.* 117 29–44. 10.1016/j.jecp.2013.08.007 24135313

[B23] Link T. Huber S. Nuerk H. C. Moeller K. (2014). Unbounding the mental number line-new evidence on children’s spatial representation of numbers. *Front. Psychol.* 4:1021. 10.3389/fpsyg.2013.01021 24478734 PMC3897877

[B24] Lonneman J. Linkersdorfer J. Hasselhorn M. Lindberg S. (2016). Differences in arithmetical performance between Chinese and German children are accompanied by difference in processing of symbolic numerical magnitude. *Front. Psychol.* 7:1337. 10.3389/fpsyg.2016.01337 27630606 PMC5005975

[B25] Ma L. (1999). *Knowing and Teaching Elementary Mathematics: Teachers’ Understanding of Mathematics in China and the United States.* Mahwah, NJ: Erlbaum.

[B26] Mark W. Dowker A. (2015). Linguistic influence on mathematical development is specific rather than pervasive: revisiting the Chinese number advantage in Chinese and English children. *Front. Psychol.* 6:203. 10.3389/fpsyg.2015.00203 25767456 PMC4341514

[B27] Miller K. F. Smith C. M. Zhu J. Zhang H. (1995). Preschool origins of cross-national differences in mathematical competence: the role of number-naming systems. *Psychol. Sci.* 6 56–60. 10.1111/j.1467-9280.1995.tb00305.x

[B28] Miura I. T. Kim C. C. Chang C. M. Okamoto Y. (1988). Effects of language characteristics on children’s cognitive representation of numbers: cross-national comparisons. *Child Dev.* 59 1445–1450. 10.2307/1130659

[B29] Miura I. T. Okamoto Y. (2003). “Language supports for mathematics understanding and performance,” in *The Development of Arithmetic Concepts and Skills: Constructing Adaptive Expertise*, eds Baroody A. J. Dowker A. (Mahwah, NJ: Erlbaum), 229–242.

[B30] Miura I. T. Okamoto Y. Kim C. C. Steere M. Fayol M. (1993). First graders’ cognitive representation of number and understanding of place value: cross-national comparisons – France, Japan, Korea, Sweden, and the United States. *J. Educ. Psychol.* 85 24–30. 10.1037/0022-0663.85.1.24

[B31] Moeller K. Pixner S. Kaufmann L. Nuerk H. C. (2009). Children’s early mental number line: Logarithmic or decomposed linear? *J. Exp. Child. Psychol.* 103 503–515. 10.1016/j.jecp.2009.02.006 19328495

[B32] Moeller K. Pixner S. Zuber J. Kaufmann L. Nuerk H.-C. (2011). Early place-value understanding as a precursor for later arithmetic performance – a longitudinal study on numerical development. *Res. Dev. Disabil.* 32 1837–1851. 10.1016/j.ridd.2011.03.012 21498043

[B33] Moeller K. Shaki S. Goebel S. M. Nuerk H.-C. (2015). Language influences number processing – a quadrilingual study. *Cognition* 136 150–155. 10.1016/j.cognition.2014.11.003 25497523

[B34] Muldoon K. Simms V. Towse J. Menzies V. Yue G. (2011). Cross-cultural comparisons of 5-year-olds’ estimating and mathematical ability. *J. Cross Cult. Psychol.* 42 669–681. 10.1177/0022022111406035

[B35] Mullis I. V. S. Martin M. O. Foy P. Hooper M. (2016a). *TIMSS 2015 International Results in Mathematics.* Chestnut Hill, MA: TIMSS and PIRLS International Study Center, Boston College.

[B36] Mullis I. V. S. Martin M. O. Loveless T. (2016b). *20 Years of TIMSS: International Trends in Mathematics and Science Achievement, Curriculum, and Instruction.* Chestnut Hill, MA: TIMSS and PIRLS International Study Center, Boston College.

[B37] Nickerson R. S. (1988). Counting, computing, and the representation of numbers. *J. Hum. Factors* 30 181–199. 10.1177/001872088803000206

[B38] Nuerk H.-C. Weger U. Willmes K. (2005). Language effects in magnitude comparison: small, but not irrelevant. *Brain Lang.* 92 262–277. 10.1016/j.bandl.2004.06.107 15721959

[B39] Okamoto Y. (2015). “Mathematics learning in the USA and Japan: influences of language,” in *Oxford Handbook of Mathematical Cognition*, eds Kadosh R. Cohen Dowker A. (Oxford: Oxford University Press), 415–429.

[B40] Perry M. Vanderstoep S. W. Yu S. L. (1993). Asking questions in first-grade mathematics classes: potential influences on mathematical thought. *J. Educ. Psychol.* 85 31–40. 10.1037/0022-0663.85.1.31

[B41] Petitto A. L. (1990). Development of number line and measurement concepts. *Cogn. Instr.* 7 55–78. 10.1207/s1532690xci0701_3 16716212

[B42] Pixner S. Zuber J. Heřmanová V. Kaufmann L. Nuerk H.-C. Moeller K. (2011). One language, two number-word systems and many problems: numerical cognition in the Czech language. *Res. Dev. Disabil.* 32 2683–2689. 10.1016/j.ridd.2011.06.004 21763104

[B43] Sasanguie D. Göbel S. M. Moll K. Smets K. Reynvoet B. (2013). Approximate number sense, symbolic number processing, or number-space mappings: what underlies mathematics achievement? *J. Exp. Child Psychol.* 114 418–431. 10.1016/j.jecp.2012.10.012 23270796

[B44] Saxton M. Towse J. N. (1998). Linguistic relativity: the case of place value in multi-digit numbers. *J. Exp. Child Psychol.* 69 66–79. 10.1006/jecp.1998.2437 9584071

[B45] Schneider M. Grabner R. H. Paetsch J. (2009). Mental number line, number line estimation, and mathematical achievement: their interrelations in grades 5 and 6. *J. Educ. Psychol.* 101 359–372. 10.1037/a0013840

[B46] Schneider M. Heine A. Thaler V. Torbeyns J. De Smedt B. Verschaffel L. (2008). A validation of eye movements as a measure of elementary school children’s developing number sense. *Cogn. Dev.* 23 409–422. 10.1016/j.cogdev.2008.07.002

[B47] Schneider M. Merz J. Sticker J. De Smedt B. Torbeyns J. Verschaffel L. (2018). Associations of number line estimation with mathematical competence: a meta-analysis. *Child Dev.* 89 1467–1484. 10.1111/cdev.13068 29637540

[B48] Siegler R. S. Booth J. L. (2004). Development of numerical estimation in young children. *Child Dev.* 75 428–444. 10.1111/j.1467-8624.2004.00684.x 15056197

[B49] Siegler R. S. Mu Y. (2008). Chinese children excel on novel mathematical problems even before elementary school. *Psychol. Sci.* 19 759–763. 10.1111/j.1467-9280.2008.02153.x 18816281

[B50] Simms V. Clayton S. Cragg L. Gilmore C. Johnson S. (2016). Explaining the relationship between number line estimation and mathematical achievement: the role of visuomotor integration and visuospatial skills. *J. Exp. Child Psychol.* 145 22–33. 10.1016/j.jecp.2015.12.004 26773209

[B51] Stigler J. Fernandez C. Yoshida M. (1996). “Traditions of mathematics in Japanese and American elementary classrooms,” in *Theories of Mathematical Learning*, eds Steffe L. Nesher P. Cobb P. Goldin G. Greer B. (Mahwah, NJ: Erlbaum), 149–175

[B52] Sturman L. (2015). “What is there to learn from international surveys of mathematics achievement?,” in *Oxford Handbook of Mathematical Cognition*, eds Kadosh R. Cohen Dowker A. (Oxford: Oxford University Press), 430–444.

[B53] Van Rinsveld A. Schiltz C. (2016). Sixty-twelve = Seventy-two? A cross-linguistic comparison of children’s number decoding. *Br. J. Dev. Psychol.* 34 181–197. 10.1111/bjdp.12122 27385154

[B54] Wong N. Y. Lam C. C. Wong K. M. P. Leung F. K. S. Mok I. A. C. (2001). Students’ views of mathematics learning: a cross-sectional survey in Hong Kong. *Educ. J.* 29 38–59.

[B55] Zhang J. Norman D. A. (1995). A representational analysis of numeration systems. *Cognition* 57 271–295. 10.1016/0010-0277(95)00674-3 8556844

[B56] Zhang J. Wang H. (2005). The effect of external representations on numeric tasks. *Q. J. Exp. Psychol.* 58A, 817–838. 10.1080/02724980443000340 16194937

